# Transforming the food system in ‘unprotected space’: the case of diverse grain networks in England

**DOI:** 10.1007/s10460-023-10535-2

**Published:** 2024-03-25

**Authors:** Stephanie Walton

**Affiliations:** Centre for Food Policy, City University of London, Northampton Square, London, EC1V 0HB UK

**Keywords:** Inclusive innovation, Grassroots movements, Food policy, Food systems, Social innovation

## Abstract

Transitioning to food systems that are equitable, resilient, healthy and environmentally sustainable will require the cultivation and diffusion of transformational sociotechnical innovations—and grassroots movements are an essential source of such innovations. Within the literature on strategic niche management, government-provided ‘protected spaces’ where niche innovations can develop without facing the pressures of the market is an essential part of sustainability transitions. However, because of their desire to *transform* rather than *transition* food systems, grassroots movements often struggle to acquire such protected spaces and so must determine how and where to generate change whilst being marginalised and exposed to unprotected spaces. The aim of this research is to gain a precise view of the multiple touchpoints of marginalisation that exist across the grassroots-government interface and to apply a new framework for conceptual analysis of these touchpoints that can help to identify where and how grassroots movements might be able to push against this marginalisation. The study finds that, by applying a ‘who, what, where’ framework of analysis to policies across this interface, it is possible to find pathways forward for achieving small wins towards food systems transformation.

## Introduction

Transitioning to food systems that are equitable, resilient, nutritious and environmentally sustainable will require transformational innovation (Herrero et al. 2020; von Braun et al. 2021; Moberg et al. 2021). The perpetual crises that are currently embedded in the way our food systems function—persistent food insecurity, chronic malnourishment, labour exploitation, environmental destruction—are not only technological in nature, but social, rooted in a set of economic assumptions and priorities that have proven ill-suited to effectively delivering the multiple outcomes needed for planet and people (Benton and Bailey 2019). As a result, transitioning to more effective and just food systems will require not only new technologies, but new forms of social organising, collaboration and exchange that are guided by a different set of values and priorities (Desa and Jia 2020; EEA 2022). Recently, there has been a growing recognition of grassroots movements as an essential source of such innovation in food systems transitions (Seyfang and Smith 2007; Smith and Stirling 2018; Sage et al. 2023).

Grassroots movements, such as alternative food networks or community food hubs, are “networks of activists and organisations that lead bottom-up solutions…that respond to the local situation and the interests and values of the communities involved” (Seyfang and Smith 2007) and where “communities have control over the process and outcomes” (Smith and Stirling 2018). Because of their rootedness in specific geographies and local communities, grassroots movements focus on solving problems and pursuing outcomes that are overlooked by the institutions typically associated with ‘innovating.’ Such institutions tend to focus on developing novel products (typically technological) within formal organisations (typically private companies) to achieve a narrow set of outcomes (typically monetary) (Edquist 1997; Fressoli et al. 2014). In contrast, as Kirwan et al. (2013) described, grassroots movements are driven to “develop alternatives to the mainstream hegemonic regime, which includes re-ordering the values and indicators of success.” They push this re-ordering through constructing and enacting social innovations, alternative ‘social practices’ (Howaldt and Schwarz 2010) that manifest a change in ‘attitudes, behaviour or perception’ (Neumeier 2012). These social innovations take shape through diverse organisational forms (e.g. cooperatives, voluntary groups) and governmental arrangements (e.g. the commons) rooted in values of ecology, equity and community health (Seyfang and Smith 2007).

The challenge for grassroots innovators is in how to effectively cultivate and diffuse their niche innovations so as to transform the systems in which they operate. Within the literature on sociotechnical transitions, governments need to provide niche innovations with resources and support to grow (Kemp et al. 2001; Schot and Geels 2008; Smith and Raven 2012; Seyfang and Longhurst 2013), but acquiring these is often contingent on being an institutional innovator and developing a technological innovation that can be easily ‘scaled up’ and ‘mainstreamed’ and won’t be too challenging to government norms and values (Smith and Stirling 2018; Ng et al. 2019). Because they are not part of mainstream institutions and their innovations are values-driven and social as well as technological, grassroots innovators operate outside the boundaries of the innovation system (Vanloqueren and Baret 2009). While some are open to ‘scaling up’ or *inserting* themselves into the mainstream even if it means adjustment or appropriation of their contributions, others are resistant to this process and are more concerned with proliferating or replicating their sociotechnical innovation within other communities to ensure their values are not erased (Fressoli et al. 2014; Hess 2013). Finally, grassroots movements typically want to *transform* systems by challenging their norms, power structures and *modus operandi*, whereas mainstream institutions want to *transition* the system through gradual changes that do not create too much political friction (Seyfang and Smith 2007; Kirwan et al. 2013; Fressoli et al. 2014; Hölscher et al. 2018; Pant 2019; GLOPAN 2020; Sage et al. 2023).

As a result, the latter group of grassroots movements struggle to get the resources they need to cultivate and diffuse their innovations and impact the wider systems (Fressoli et al. 2014). These resources and support are a crucial pre-requisite to cultivating an innovation so that it can proliferate and ultimately change or displace a *regime*, but getting said resources to displace a regime is difficult when it is the regime that controls the resources (Vanloqueren and Baret 2009). As LaForge et al. (2017) has shown, grassroots innovators may be provided with some resources—such as, for example, the UK government’s £4 million *Community Research Networks* fund (UKRI 2023a)—but only up to the point where their social objectives begin to challenge incumbent institutions and power structures.

Thus a crucial question emerges as to how grassroots innovators can impact the broader system whilst they are so under-resourced and marginalised (Köhler et al. 2019). This is a question of power and empowerment—a subject that is gaining increasing attention within transition studies (Raj et al. 2022). Recent advances in this area re-casts grassroots innovators as political actors with agency rather than technologists looking for government patronage (Hess 2013; Schreur, 2016; LaForge et al., 2017; Marletto and Sillig 2019; Clark et al. 2021; Gregg et al. 2020). Implicit in such re-framings is that there could be multiple policy arenas in which grassroots movements could assert their agency—e.g. social policy, food policy, health policy, rural affairs, industrial policy, etc.—not only innovation policy. Indeed, there is a growing recognition among transition scholars of the need to not only look at singular innovation policies but policy mixes (Geels 2019, 2020). The importance of looking across policy mixes is echoed among agricultural innovation systems (AIS) scholars who are calling for a new focus on *innovation ecosystems*, recognising that innovation is cultivated and diffused within a system of overlapping systems rather than through linear processes of technology diffusion (Pigford et al. 2018; Klerkx and Begemann 2020; Payne-Gifford et al. 2021). Such a perspective requires moving beyond innovation policy to look at the much wider range of policies that sit at the interface between grassroots innovators and government. However, a process for mapping the various touchpoints on this interface—and a framework for conceptual analysis to interpret them—has not yet been developed.

The aim of this research is to gain a precise view of the multiple touchpoints of marginalisation that exist across the grassroots-government interface and to apply a new framework for conceptual analysis of these touchpoints that can help to identify where and how grassroots movements might be able to push against this marginalisation. I focus on two research questions to achieve this aim: (1) What are the touchpoints where grassroots innovators are impacted by government policy and (2) How do these touchpoints marginalise grassroots innovators and limit their abilities to cultivate and diffuse their innovations? I answer these questions using a case study—diverse grain networks and national policy in England—which allows for an exploration of the intricacies of grassroots marginalisation that can take place for a specific sociotechnical innovation. Whilst there are intricacies that are no doubt case specific, grassroots movements have been found to encounter a similar set of challenges in diverse settings, so this case study will have relevance beyond this particular study. What follows is, first, a review of the literature on grassroots innovations and the introduction of Schillo and Robinson’s (2017) ‘who, what, why’ framework proposed for conceptual analysis. The results from iterative policy mapping conducted through in-depth interviews with grassroots innovators are then presented to identify these touchpoints. Finally, these touchpoints are analysed within Schillo and Robinson’s (2017) framework to identify the specific dimensions of marginalisation and explore potential pathways forward through them.

## Unprotected space and the grassroots-government interface

Research on how to effectively cultivate and diffuse grassroots innovations has drawn primarily on strategic niche management (SNM) theory and the “multilevel perspective” (MLP). MLP is a framework for understanding how sociotechnical transitions occur, with much of its focus placed on the role of niches—small and emerging pockets of socio-technical innovation that operate on the boundaries of the dominant socio-technical regime (Geels 2002). SNM is concerned with how to manage and develop these niches which, if adequately nurtured, could alter or replace the regime (Kemp et al. 1998). A core tenant of SNM is that niches must be given a ‘protected space’ in which to learn and develop free from the selection pressures of the market (Kemp et al. 2001; Smith and Raven 2012; Raven et al. 2016). Once the niche innovation has matured, protective measures can be removed and the innovation can navigate its external environment in its own right. Seyfang and Smith (2007) drew heavily on this theory in their seminal work on grassroots innovations, describing them as *innovation niches* that, if taken seriously and properly supported, have the potential to transform the dominant regime. They proffered that, if armed with knowledge of how grassroots niches emerge and operate, it is possible to make policy recommendations for how to support them within a protected space until they are ready to go forth and re-shape the regime.

Rooted in this framing, much of the subsequent research on grassroots niches has focused on studying the specific challenges they encounter in the development of their innovations (Hossain 2018). Several studies on the inner workings of grassroots movements reveal the immense difficulty in acquiring resources, developing internal infrastructure and capacity, navigating a plurality of ideas, values and politics among members, communicating and reporting on progress, fending off co-option by larger institutions and maintaining the will and (wo)manpower to drive efforts forward (Seyfang and Longhurst 2013; White and Stirling 2013; Kump and Fikar 2021). From this research, subsequent policy recommendations have focused on what policymakers need to know about grassroots movements when providing them a protected space to ensure that their efforts towards supporting them are effective—creating open learning environments, building capacity, provide opportunities for emergent knowledge exchange, etc. (Seyfang and Haxeltine 2012; Kirwan et al. 2013; Smith et al. 2014; Hoffecker 2021; Grandadam et al. 2022; Ng et al. 2022). The pre-supposition, however, is that governments will create a protected space for grassroots innovations (Schot and Geels 2008; Smith and Raven 2012; Raven et al. 2016).

However, the centrality of the provision of protected space poses a problem for grassroots movements because it implies that, until they have been granted such a protected space, they are limited in what they can achieve. Even more critical than this is the implication that grassroots movements are not empowered or lack power to generate change until a government supplies them with it via a protected space. In this framing, grassroots movements have a certain degree of agency to advocate for themselves to acquire a protected space, but this is distinct from the empowerment they would receive once they get it (Smith and Raven 2012). The outcome is that grassroots movements must put their limited resources and energies towards getting policymakers to give them a protected space.

Since protected spaces provide power, securing such a space is inherently a political process—one in which grassroots innovations are competing with other innovations as well as industry incumbents in a battle of narratives, coalition-building, public support and government lobbying (Smith and Raven 2012; Hess 2013; Scoones 2016; De Schutter 2017). Within this political dogfight, grassroots movements are inherently at a disadvantage. This is not, however, necessarily because they are under-resourced compared to their competitors (although this is certainly a challenge), but because what grassroots movements want is the power to *stretch-and-transform* the regime rather than simply *fit-and-conform* within it. This distinction, made by Smith and Raven (2012), is akin to the difference between calling for a food systems *transformation* as opposed to a food systems *transition—*the latter tends to be more palatable to those within the regime whilst those on the margins surrounding the regime are calling for the former. Government institutions may be willing to extend some support, but only insofar as grassroots movements are willing to fit and conform rather than stretch and transform (Smith and Raven 2012). These protective space ‘mirages’ essentially gaslight grassroots movements by making it appear that government is creating a protected space but without actually empowering grassroots innovators in a meaningful way (LaForge et al. 2017). The end result for those working within these grassroots movements is an overall feeling of marginalisation and disempowerment.

Some scholars have begun to explore a different conceptualisation of power and empowerment within grassroots innovations (Raj et al. 2022), outlining the power that grassroots innovators hold before it has been granted to them by government and, subsequently, what they might be able to do with this power (Klerkx et al. 2010; Hess 2013; Schreuer 2016; Laforge et al. 2017; Marletto and Silig, 2019; Clark et al. 2021; Gregg et al. 2020). This approach paints grassroots innovations as social movements rather than innovation niches (Gregg et al. 2020), allowing for a shift in focus away from securing a protected space to pushing for broader social change. Grassroots movements can make strategic choices about where to direct their resources and assert their power to successfully stretch and transform their environment—where to achieve ‘small wins’ that have transformative potential (Bryson 1988; Termeer and Metze 2019; Bours et al. 2022; Weick 1984).

Effectively and efficiently utilising limited resources towards a stretch-and-transform agenda requires thorough knowledge of (1) all of the touchpoints on the grassroots-government interface and (2) if and how those touchpoints marginalise them and their innovation and thus need to change (Ganz 2004). Acquiring this knowledge requires moving beyond analysing policy through a dichotomous lens of “Is this creating a protected space for us or not?” and pushing to deeper levels of analysis of the grassroots-government interface. While a substantial amount of scholarly work has been done on the *lack* of policies in place to support grassroots movements, a framework has yet to be introduced for conducting a conceptual analysis of the *existing* touchpoints on the grassroots-government interface that reveals potential small wins.

Schillo and Robinson (2017) propose a ‘who-what-why-how’ framework for analysing if policies are inclusive of a broad range of people, technologies and values. The ‘*who’* is concerned with which groups of people should be included and whether they are included effectively. The ‘*what’* is concerned with the types of innovation that are included. The ‘*why’* is concerned with expanding the expectations of what innovation is meant to deliver beyond economic growth to include a much wider range of health, social and environmental outcomes and that the benefits from achieving those outcomes are equally distributed. Finally, the *‘how’* is concerned with the governance mechanisms that will make innovation more inclusive. Governance is undoubtedly a crucial component to sustainability transitions (Patterson et al. 2017; Smith and Stirling 2018; Clark et al. 2021; Galli et al. 2020; de Boon et al. 2022) that was unfortunately beyond the scope of this research which is focused on examining existing policies rather than policymaking processes. Thus, I apply an augmented ‘who-what-why’ framework of policy analysis to the questions of if and how a policy is marginalising grassroots innovators.

Bringing such a framework to bear on all of the touchpoints on the grassroots-government interface can shed light on alternative touchpoints where grassroots movements might focus their limited resources without first securing a protected space. Such an analysis could, in itself, empower grassroots movements with knowledge and intentional directionality towards stretch-and-transform actions by applying more analytical precision to the problem of marginalisation. As such, the following analysis is focused on the technical aspects of policies, not to avoid politics but to enact politics through the utilisation of knowledge met with agency.

## Method

### The case study: diverse grain networks as a grassroots innovation

One of the major challenges currently facing food systems is the erosion of plant genetic diversity and, with it, food systems’ resilience and adaptability to environmental change (FAO 2010, 2019; Jones et al. 2021; Khoury et al. 2022). Since the Green Revolution in arable farming, plant breeders, agronomists and policymakers have focused almost exclusively on breeding high-yielding varieties that respond well to synthetic fertilisers (Benton and Bailey 2019). In addition to creating a genetic bottleneck (Voss-Fels et al. 2019), the widespread diffusion of high-yielding grain varieties has contributed to a societal shift towards larger, industrialised monoculture farms and the decline of small mixed farms and organic and agroecological (O-Ae) systems. This was accompanied by the institutionalisation of plant breeders’ rights which drove a decline in farmer seed saving and seed exchange worldwide (Shiva 1996; Kloppenburg 2010).

In England, these sociotechnical reconfigurations in farming coincided with the invention of the Chorleywood bread process which enabled the industrial manufacturing of bread (BBC 2011). To achieve the desirable ‘fluffy white loaf’ through industrial baking processes requires wheat that meets a narrow range of ‘specifications,’ the most important of which is high levels of protein—levels not typically achieved without the application of synthetic fertilisers (Hawkesford 2014). Industrial bakeries began working with plant breeders to ensure their input specifications were factored into plant breeding and agronomic strategies (Meynard et al. 2017; Magrini et al. 2019), further narrowing plant genetic diversity and necessitating the use of synthetic inputs.

While organic horticulture has been slowly growing, organic arable farming has been slower to take off due to the ‘lock-in’ of the existing breeding-farming-processing networks to high-protein high-yielding wheat varieties (Lammerts van Bueren et al. 2011; Meynard et al. 2017; Magrini et al. 2019). However, there has been a growing interest at the grassroots level in cultivating new sociotechnical systems around grain, flour and bread that prioritise resilience, ecological health and equitable value chains. In England, grassroots plant breeders have been working to introduce wheat varieties that do well in O-Ae farming systems and rely on genetic diversity for resilience. Some breeders focus on the development of cross-composite populations—ultra-diverse grains that are the product of crossbreeding numerous varieties together, the opposite of a monoculture variety (Döring et al. 2011; Wolfe and Ceccarelli 2020). Others focus on tracking down dis-used varieties that were popular prior to the onset of synthetic inputs and re-introducing them onto farms, commonly referred to as ‘heritage grains.’^1^ Others are pursuing a mixture of both. While the efforts themselves are diverse and diffuse, the outcome is a collection of genetically diverse grains specifically intended for O-Ae farming systems—a grassroots technological innovation.^2^ Grassroots plant breeders argue that these grains will be incredibly important for sustainable food systems because they do not rely on synthetic inputs and because their in-built genetic diversity contributes to greater yield stability over time and resilience against unpredictable external stressors in the face of future environmental change. An emerging body of academic research is beginning to argue for the importance of these grains as well (Østergård et al. 2009; Döring et al. 2011, 2015; Wolfe and Ceccarelli 2020).

Because they are bred for diversity and O-Ae farms rather than industrial processing, these diverse grains frequently do not meet the specifications required by industrial bakeries. So, to diffuse their technological innovation, grassroots breeders and farmers found bakers willing to work with unfamiliar flour and who shared similar values of resilience, ecological health and equity. These values are rooted in the norms and values of the much broader movement towards creating alternative food networks (AFNs) as a substitute for industrial food systems (IFSs). Whilst IFSs are rooted in a ‘productivist’ paradigm focused on the maximisation of efficiency and yields (Benton and Bailey 2019), AFNs promoted an ‘ecological’ paradigm, placing a high value on agricultural systems that work with nature and within planetary boundaries (Harris 2009; Sarmiento 2017; Mert-Cakal and Miele 2020; Sage et al. 2023).

As more bakers came on board, more farmers started to grow diverse grains and sell them directly to bakeries where they could negotiate a fair price rather than selling into the commodities market where they have no say over price. As interest has grown, breeders, farmers, millers and bakers have coordinated to form local, regional and national networks for facilitating knowledge generation and exchange, participating in collaborative breeding programmes and constructing new social provisioning models and modes of exchange with their suppliers and customers (Sage et al. 2023). Compelling new social relationships are being formed between those who typically never collaborate across grain supply chains (Mulgan 2007) and new forms of collective ownership are being developed. The result is a slowly emerging ‘collaborative economy’ (Kostakis and Bauwens 2014) where food producers at the grassroots level are co-producing new values-led social reconfigurations around a novel technology.

### Data collection methods

To identify the relevant policies and understand how they intersect and interact with diverse grain networks, a novel method of policy mapping was developed that combined interviews and iterative semi-participatory policy mapping, drawing on emerging methods in participatory food systems mapping (Jacobi et al. 2019; Valette et al. 2019) with the added layer of placing policies on top of systems processes.

#### Recruitment

To aid in the development of and coordination of diverse grain networks, leaders in diverse grain networks have developed a map of the farmers, millers and bakers who are working with these grains (https://www.ukgrainnetwork.com/). Because the focus of this study was on national policy and to ensure a sufficient data sample could be collected, everyone on this map was contacted to participate in this study rather than focusing on a specific geographic area. As the study progressed, a snowball method led to the addition of others who work with but are not on the map. 23 people in total participated in the study covering a range of activities within the network (Table 1).

**Table 1:** Number of participants that do a specific activity. Several participants were active in more than one area

Activity	*n*
Breeding/restoring from gene banks	7
Farming	9
Milling	6
Baking	9
Network coordination	3

#### First round of interviews

Policies were identified through two rounds of unstructured interviews conducted between July 2021 and May 2022*. *Formal discussions were supplemented by informal discussions and conferences hosted by food producers. The first interview with each food producer lasted approximately 2 hours. Participants were asked to describe the origins of their business, how they started working with diverse grains, their production process and the hurdles they encounter as part of the day-to-day of working with a new sociotechnical innovation. The interviews were coded into three levels: (1) the stage in the value chain, (2) the hurdles encountered and (3) policies that were explicitly mentioned in relation to hurdles.

#### Policy review

Where policies were explicitly mentioned by participants, they were then searched for on government websites and the relevant materials were aggregated. Where participants did not explicitly mention a policy but discussed a hurdle, relevant policies were then searched for that could potentially intersect with that hurdle. For example, several bakers discussed the difficulty of finding skilled bakers to hire, but no bakers mentioned a specific policy related to this. This led to searches on government websites for material related to worker training and skills development from which policies and programmes that might be relevant to this hurdle were then collected.

Whilst local policies are incredibly important in the cultivation of alternative food networks, the focus of this study was only on national policies, so only national government websites were searched. A focus on national policies was chosen because, first, national policies are the primary focus within the strategic niche management and sustainability transitions literature and, second, because while local policies are an important source of support, most of the major policies that effect farmers (e.g. subsidies, environmental regulations, food safety) and food producers (e.g. taxes) are established on a national level.

#### Second round of interviews

In the second round of interviews, participants were taken through the list of policies relevant to their stage of the value chain. They were asked to discuss if and how specific policies affected their operations. These interviews were then used to refine the coding from Round 1 to remove or include policies. To be included, a policy had to directly impact or have the potential to impact a food producers’ ability to overcome a hurdle they encounter in their day-to-day. Policies and hurdles only had to be relevant to one participant to be included. The results are an aggregation of food producers’ experiences rather than representative of the whole sample.

### Data analysis

#### Policy coding

Once a final list of policies was determined, each policy was then coded according to how participants described the way the policy intersects with a hurdle and were categorised into three groups: helpful policies, hindering policies or mismatches. Helpful policies are those that provide resources or support in overcoming a hurdle. Hindering policies are those that either prevent food producers from overcoming a hurdle or create a hurdle themselves. Mismatches are those policies that have the potential to be helpful but are not by some aspect of their design.

#### Analysis of marginalisation dimension

Once policies were coded, discussions of the mismatched and hurtful policies were then analysed for the specific dimensions against which participants said the policies were marginalising. Policies were then categorised according to the ‘who’, ‘what’, ‘why’ categorical framework.

## Results

All hurdles and policies are shown in Table 2. Hurdles are sorted according to where in the value chain they are encountered. Each hurdle and the associated policies are described below, beginning with baking (bottom row) and moving backwards along the value chain towards network development (top row).

**Table 2:** A map of the relevant policies that effect food producers in their work with diverse grains, categorised according to the hurdles they intersect with (rows) and whether they are helpful, a hindrance or a mismatch (columns)

Value Chain	Hurdles	Helpful	Mismatch	Hindrance
Network	Funding for network development	- Food Systems Transformation Fund (subject) - National Lottery Community Fund (subject)	- Food Systems Transformation Fund (scale) *- *National Lottery Community Fund (application/reporting)	
Inputs	Funding for R&D	- Horizon 2020 - Defra non-competitive grants - Participatory research funding	- Farm Innovation Programme - R&D Capital Allowance	
	Selling seeds	- Defra non-competitive grants		- The Seed (National List of Varieties) Regulation (2001) - The Seed Marketing Law (2011)
Farming	Unstable tenancies			- Agricultural Tenancies Act (1995)
	Environmental standards		- EU Regulation 834/2007 on organic labelling and marketing	
	Farm Labour			- National Planning Policy Framework
Post-harvest processing	Post-harvest infrastructure	- Basic Payment Scheme - Countryside Stewardship (landowners) - Annual Investment Allowance	- Farming Investment Fund - Added Value Grant - 130% Super-deduction - Countryside Stewardship (tenant farmers)	
	Fortification			- Bread and Flour Regulation (1998 (fortification)
Baking	Marketing		- The Bread and Flour Regulation ﻿^1998^ (labelling)	
	Unstable tenancies			- Landlord and Tenant Act (1954)
	Skills and training (Baking***)***	- Apprenticeships	- Apprenticeships	

### Baking

#### Skills and training

Bakers raised that finding people with experience—or an interest in gaining experience—in working with diverse grains was a challenge. Flours from diverse grains perform differently than standard flour. The variability of composition and consistency of flour that results from the genetic diversity requires a heightened degree of attentiveness from the baker and a willingness to constantly adapt formulations. But most bakers—even those working in craft or artisanal bakeries—are trained to work exclusively with standard flour.

Two bakeries said that government-funded *apprenticeships* were helpful for filling the skills gap. However, other participants expressed an openness and interest in taking on apprentices but said that they were not familiar with the process so had not pursued it. Others said they felt it was risky to hire inexperienced staff and that they did not have the capacity to train someone—a common challenge for small businesses.

#### Urban commercial rents

Some participating bakers who ran small bakeries in cities encountered difficulties with unstable tenancies related to rent increases. As data was being collected for this study, inflation began to take off and the costs of doing business were rising—particularly energy. However, rent was the item raised as a particular problem. One participant reported facing a 50% rent increase after the property was sold from the government to an international investment bank.

While residential rents were regulated prior to the 1980s, commercial rents have not been regulated since the Landlord and Tenant Act (1954) leaving rent negotiations up to the landlord and the tenant with no government involvement. As property values rise, pressure is placed on the business to maximise their margins to stay competitive. When rent does go up, businesses can pass the costs on to their customers and/or reduce other input costs. In the case of the participant above, they chose to raise prices but said they might have to reduce their use of diverse grains if things get worse in the future.

#### Marketing ‘heritage bread’

To mitigate the difficulty of working with flour from diverse grains, most of the participating bakers reported using a small percentage in their loaves—between 10% and 30%, but some used it for as little as 3% (two participants used diverse grains for 100% of their flour). While bakers said this was necessary to produce the types of loaves they said their customers want, others along the value chain—particularly farmers—took issue with this approach.

*They’ve bought some white flour from somewhere… and then they’re holding the 10% of local grain with a name and then that’s what’s selling the loaf and that’s the narrative of the bakery. That’s not cool. A, because it’s like a bit disingenuous and, B, because it doesn’t actually facilitate the change on farm because farmers want to grow the 90% not the 10%.*

Some farmers were concerned that this lack of protection on the marketing value of bread made from ‘local’ or ‘heritage’ grains puts those who would use a higher percentage at a disadvantage and disincentivises using more. The flour is expensive and the more a bakery uses in a loaf, the more it eats into their margins. Competitors can take advantage of the marketing value of terms like ‘heritage’ but with lower input costs, opening up the risk of participants being absorbed and diluted by larger competitors.

Some farmers suggested there should be some type of labelling or certification standard that would guarantee transparency for the end customer and protect the marketing value for those who are using more flour. The Bread and Flour Regulations (1998) place labelling restrictions on bread, but this is only in relation to the term ‘wholemeal’ (which several participants pointed out is quite easy to bypass).

### Post-harvest processing

#### Fortification

The Bread and Flour Regulations (1998) requires millers who are producing non-wholemeal wheat flour to fortify it with folic acid, iron, niacin and thiamine. Participants said this presented a major financial and logistical challenge. Others voiced concern that requiring small mills to fortify poses a risk to consumers because small mills do not have the equipment to mix the additives in properly. Some millers found it so difficult to meet the requirements that they stopped milling non-wholemeal flour entirely and only produced 100% wholegrain flour, cutting off a major source of income as white flour sells in much higher quantities than wholegrain flour.

#### Investing in the physical infrastructure

When selling grain as a commodity, grain cleaning, drying, storage, testing and transport is typically handled by grain merchants and large mills who work with enormous quantities of grain and use suitably enormous processing machinery. Because producers growing diverse grains do not want to sell their grain as a commodity, they cannot take advantage of existing physical infrastructure. As a workaround, farmers are acquiring the machinery to do these post-harvest steps themselves.

Farmer participants said they relied either on bank loans or personal funds to finance grain dryers, cleaners and mills. One farmer also said that, since they can now set the price of their grain by selling directly to bakers, they no longer rely on farm subsidies to prop up their business. Now, they can invest the subsidies they receive through the *Basic Payment Scheme* (Rural Payments Agency and Defra, 2023) and *Countryside Stewardship* (Rural Payments Agency et al. 2023) into other parts of their business. A second farmer also said that a shift is taking place among farmers towards seeing subsidies as sources of farm investment rather than a salary. While farmers expressed that there are many flaws with these subsidy schemes, all of them (except for the tenant farmer, see below) received both subsidies and so had access to those funds.

Farm machinery grants were another potential source of capital. The *Added Value Grant* is meant to help farmers acquire machinery to “process, diversify and add value” to their products (Defra 2022). While this grant could not be put towards grain cleaning or drying equipment, it could be put towards the purchase of a mill. However, farmers described a mismatch because, in addition to the time required to apply, the grant amounts are too big for the scale at which these farmers are operating and only covers 40% of the cost of an item. Also, because grants are paid in arrears, farmer need to have the 100% of funds available up front to take advantage of the grant.

*It seems to be very impractical that you have to have a lot of money to be able to get the grant and if you got the money you don’t need the grant.*

More traditional farm machinery grants, such as the *Farming Equipment and Technology Fund* within the *Farm Investment Fund* (Rural 2021), were similarly mismatched for the same reasons as well as because these machinery grants are applicable only to a pre-selected list of machines which is determined by Defra and which farmers have to buy new rather than second-hand. Post-harvest processing machinery is not included on this list. So these grants are mismatched to the needs of these farmers and the resources and cash flow necessary to fund the acquisition of the grant.

Tax deductions were also raised in relation to financing machinery. Farmers mentioned the *Annual Investment Allowance (AIA)* (Treasury 2022) as helpful—a 100% tax deduction on the cost of any new or used machinery purchased for the farm. However, they pointed out that they are not able to take advantage of a *130% Super-Deduction* (Treasury 2021) on large capital investments because this is only available to businesses that pay corporation tax. These farmers are registered as sole traders or partnerships so they cannot take advantage of this scheme.

### Farming

#### Farm labour

Mixed-use farms, which O-Ae farms tend to be, are more labour-intensive to run than if growing monocultures. Some farmer participants described relying on family, friends or contractors to help on the farm, but multiple farmers discussed the inability to house someone on their farm—a standard practice—as one of the main hurdles to hiring help. Building accommodations is restricted by the *National Planning Policy Framework (NPPF)* (MHCLG 2021) that prevents building on protected land. While there is a provision in the NPPF that allows for building farmworker dwellings, it requires proving that it is essential to the operations of the farm that a worker be present 24 h a day. This is typically only the case for livestock farms, so it is very difficult to get planning permission for arable farms.

#### Ensuring environmental quality

Some farmers were also concerned that competing farmers could grow diverse grains with synthetic inputs and claim the same premium as O-Ae farmers.

*The [supermarket] deal, in theory, is interesting and, in theory, is good. They are paying £650 a tonne. But there is no restriction on the growing being organic or regenerative or anything like that…. Instead, these people this year who are now growing for [this supermarket] and being promised £650 a tonne off the combine can use whatever pesticides and fertiliser they want to grow that crop.*

Up to this point, diverse grains have only been of interest to O-Ae farmers in need of varieties that would perform well without inputs. But as the marketing potential of terms like ‘heritage’ grows, interest may also grow among non-O-Ae farmers who can use inputs to boost their yield, making it more difficult for O-Ae farmers to find buyers.

The only law in England that regulates environmental standards for food is the *Council Regulation (EC) 834/*(2007)* on organic labelling and marketing* which protects the marketing value of the term ‘organic’ by prohibiting its use except for producers who are certified. However, this law does not ensure that certain seeds, crops or varieties are only to be produced to a certain environmental standard. Organic farmers are not allowed to use non-organic seeds without special derogations, but non-organic farmers can use organic seeds if they wish.

#### Short-term agricultural tenancies

Participating farmers were a mix of both land-owning and tenant farmers. Because of the investment required to process diverse grains—and the substantial risk involved—tenant farmers said it was much more difficult to start growing diverse grains than landowning farmers.

*If you have a ten-year lease, if you have a five-year lease, you’re not thinking long term because you can’t. You can’t invest in something that you’re going to be able to hand down to your grandchildren…. The most simple thing you can do to make things more sustainable in terms of land use and ecological decision-making is giving longer leases.*

Prior to the *Agricultural Tenancies Act *(1995), it was common for tenant farmers to get lifelong tenancies and have strong protections against evictions. The 1995 act removed these protections by removing government regulations on tenancy agreements and, as with commercial real estate, leaving negotiations to be worked out privately by landowners and tenants. As this effectively opened up agricultural tenancies to wider land market conditions, the outcome for tenants has been much shorter tenancies. The average in 2021 was 5 years 8 months (Defra 2023a). For some O-Ae farms, this is shorter than the length of a full rotation. If a farmer has no guarantee that they will be on the farm for long, their risk exposure from any investments they make on the farm is greater.

Tenant farmers were also unable to access the *Countryside Stewardship Scheme* and receive subsides for environmental management (although this is in the process of changing). These schemes require a 5-year commitment and there is no flexibility once they have been agreed to. This makes it impossible for tenant farmers on shorter tenancies to participate or benefit from the cash transfers that they could invest in other parts of the business, such as landowning farmers are able to do.

### Inputs

#### Selling seeds

The hurdle that loomed largest for all participants was the illegality of selling diverse seeds. For a variety to be sold to farmers, it must first be placed on the National List of Varieties (The Seed Marketing Regulation 2011). To be placed on the National List, a variety must go through a series of tests that prove it is distinct, uniform and stable (DUS) (The Seeds (National List of Varieties) Regulations 2001). DUS is meant to ensure that a variety has one or more identifiable characteristics that set it apart from others on the list (distinct), is genetically identical in its important characteristics (uniform) and it will maintain those characteristics after successive plantings (stable). DUS is the basis on which plant breeders’ rights are assigned by making it possible to clearly distinguish one variety from another.

Diverse grains do not adhere to the DUS framework because, first,  they contain multitudes of varieties so they must be identified at a population level rather than according to uniformity from one individual variety to the next. Second, they aren’t uniform—they express a range of characteristics. Third, they aren’t stable—they change from year-to-year and farm-to-farm. For these reasons, diverse seeds cannot technically be placed on the National List and therefore it is illegal to sell them. This left participants to navigate a complex regulatory environment. Most made the argument that you are able to sell grain for milling, but not for seed and that once it leaves their farm, they are unaware of what happens to it. Nevertheless, all participants considered these laws to pose a major barrier to diffusing seeds.

However, participants had been able to source funding from an NGO to hire a lawyer and actively lobby government to institute policy change to make it legal to circulate diverse seeds. Participants were then able to get funding directly from Defra through a *non-competitive grant* to present their research on the benefits of diverse seeds and a customised system for how to track the circulation of seeds between farmers. These efforts were successful and led to the institution of *The Seeds Marketing (Hetergenous Material) (Temporary Experiment) (England) Regulations* (2023).

#### Funding for R&D

Breeding any new variety is a resource-intensive process. Typically, plant breeding is funded through the royalties from acquiring plant breeders’ rights. However, even if it were possible to get plant breeders’ rights on non-DUS varieties, these participants expressed little interest in acquiring them.

*My aim in our breeding project on the farm is not to patent any of the seeds or profit from any of the crosses and populations that we build from them. Rather, it’s to share with the network at cost [of what would be paid if sold as grain] and for the network to effectively, as a community, share ownership of them as it were.*

As a result, the development of diverse grains has been and is dependent on public funding and/or the voluntary efforts of individuals. Most diverse grains in circulation have been developed through individuals donating their time and resources. Participants pointed out that relying on the donated time and resources of individuals limits breeding efforts only to what they can donate. It also means that only those who have resources to donate (e.g. land, other income sources) are able to do it.

Without any anticipated income from plant breeders’ rights, this makes public funding essential for the development of diverse grains. The YQ population was developed by the Organic Research Centre (ORC) through a *non-competitive Defra grant* (Defra 2001, 2020) and subsequent *Horizon* 2020 projects. One participant discussed getting funding through a *Participatory Research Grant* (Research England 2022) for a participatory breeding project by partnering with an academic at a university. However, aside from small one-off projects, public funding for breeding has been in decline in England for many years. New funding has been made available through the *Farming Innovation Fund* (UKRI and Defra 2021) for R&D that will deliver on environmental objectives. However, as with machinery grants for farmers, participants discussed multiple barriers to applying for and winning these grants, first among them being that there is very little perceived interest from UKRI in developing diverse grains, participatory breeding programmes or in O-Ae farming methods. There was also the expectation among participants that their work would not fit with UKRI’s expectation that whatever comes of the research must generate economic value.

*Because we’re very clear that the output is a common rather than something that can be patented, that doesn’t sit all that well often with a lot of this kind of programs necessarily where the innovation would be something that could be, you know, generate capital in and of itself.*

Even if participants were interested in commercialising outputs, participants mentioned that current seed marketing laws cut off any routes to monetary gain for non-DUS seeds—putting funding bids for such projects at a disadvantage. Also, applying for funding like this requires being part of multi-organisational research collaborations. While formal research institutions have the capacity to develop research partnerships, such partnerships are difficult for individuals who are not based in research institutions to develop.

In addition to the mismatches around funding, there was also a mismatch on taxes. The *R&D Capital Allowance* allows a 230% tax deduction for SMEs on R&D-related expenditures – of which plant breeding is included (HMRC 2016). However, as with the 130% Super-deduction, this relief only applies to business paying corporation tax which most farmers are not.

### Network

#### Funding for network development

Advancement activities such as coordination and organising, educational events and knowledge exchanges, non-breeding related research (e.g. nutritional density of diverse grains), strategy development, community engagement and political lobbying are also a challenge to find funding for. Participants said that what external funding they do get comes from NGOs or private individuals.

Two sources of relevant government funding were identified—*National Lottery Community Fund* (National Lottery, 2023) and the *Food Systems Transformation Fund* (UKRI 2022a). The former is the primary funder for community development projects in England and two participants described winning National Lottery grants to develop community bakeries and work with schools. However, one participant said they would not apply again due to the difficulty of the application process and the reporting required once the grant was received. Therefore, this policy has been labelled as both helpful and a mismatch in that it is relevant to the advancement activities of the diverse grains network, but the burdensome application and reporting requirements are a disincentive for pursuing it.

The *Food Systems Transformation Fund* was a major UKRI research fund launched for large research partnerships that integrate natural and social sciences. One participant did apply for this grant in partnership with several academic institutions but was unsuccessful. However, this grant was categorised as helpful because the subject of the grant was relevant to the food producers and so could have been helpful. Also, unlike the *National Lottery Community Fund*, the requisite partnerships with universities meant that food producers didn’t have to bear the full burden of the application process.

## Analysis and discussion

The aim of this research was to gain a precise view of the multiple dimensions of marginalisation that take place across the grassroots-government interface. Now that the various touchpoints across this interface have been mapped, these touchpoints are analysed below using a who-what-why conceptual framework (Schillo and Robinson 2017) to consider where grassroots movements might stretch and transform the regime when they haven’t been provided with a protected space.

Applying Schillo and Robinson’s (2017) who-what-why framework (excluding the ‘how’ for the purposes of this study), Table 3 lists the policies that are mismatched or a hindrance to the efforts of the diverse grains network and outlines the precise dimensions against which these policies marginalise participants. The ‘who’ column indicates where policies marginalised participants based on one of their roles or characteristics. As is true of grassroots innovators more broadly, participants wore many hats. In this column is named which of these hats is marginalized by that specific policy. The ‘what’ is linked to innovations that participants are cultivating, both social and technological. Again, several social and technological innovations are being cultivated by participants but this column lists only those against which marginalisation was described in relation to a policy. The ’why’ describes the specific values and desired outcomes that policies marginalise.

**Table 3:** The targeted dimensions of marginalisation of individual policies across the who-what-why framework

Policy	Who (roles and characteristics)	What (innovations-social and technological)	Why (values and desired outcomes
Farm Innovation Fund	Small farms grassroots innovator	Heterogeneous seeds seed commons participatory breeding	Common ownership equity
Countryside Stewardship (tenant farmers)	Tenants		
Agricultural Tenancies Act (1995)	Tenants		
Landlord and Tenant Act (1954)	Tenants		
National Lottery Community Fund (application/reporting)	Small organisations		
Farming Investment Fund	Small organisations		
Added Value Grant	Small organisations		
Apprenticeships	Small organisations		
Bread and Flour Regulation (1998) (fortification)	Small organisations		
130% Super-deduction	Sole trader		
R&D Capital Allowance	Sole trader		
National Planning Policy Framework	O-Ae arable farmers		
The Seed (National List of Varieties) Regulation (2001)		Heterogeneous seeds	
The Seed Marketing Law (2011)		Heterogeneous seeds	
EU Regulation 834/2007 on organic labelling and marketing			Ecological
The Bread and Flour Regulation (1998) (labelling)			Ecological

The *Farm Innovation Fund* is the only policy where participants described being marginalised across the three dimensions—the ‘who’, the ‘what’ and the ‘why.’ The fund is the type of policy that is commonly the focus of discussions within agri-innovation systems and innovation and transition studies—it is meant to support a sustainability transition but ends up being exclusionary. The application process is gruelling and time-consuming, making it difficult for time-constrained small- and mid-sized farmers and organisational leaders to apply (despite UKRI’s and Defra’s attempts to be inclusive of micro-enterprises). It is also a requirement that several organisations and types of businesses apply together but building such partnerships is time-consuming and, more importantly, dependent on larger institutions taking an interest in the subject matter. Another crucial challenge is that grants are paid in arrears. This is particularly concerning for smaller organisations that do not have large capital stocks available to fund staff costs or large purchases up front (Ng et al. 2019). UKRI is transitioning to funding all of their grants in arrears (UKRI 2022b; UKRI 2023b), which raises significant concerns about the growing exclusivity of accessing innovation funds for smaller organisations, not only grassroots ones. Finally, there was also the strong feeling held by participants that UKRI and Defra would have little interest in the development of seeds for which the primary aim is not yield and that there would be discomfort with the social innovation of a seed commons. Underneath these, participants described a mismatch between their values of equity and common ownership and the prioritisation of productivity and economic returns held by UKRI and Defra. All of these dynamics effectively exclude grassroots innovators from funds that are meant to be the primary drivers of the agricultural transition in England.

While the *Farm Innovation* Fund marginalised participants across the three dimensions, the other policies were more specific in their points of marginalisation.

### Who (roles and characteristics)

The precise intersections between the several policies and the ’who’ shows that, while innovators in the diverse grains network are marginalised across a range of policies and domains, very little of this marginalisation was based on participants’ role as grassroots innovators. Rather, it was more common for participants to be marginalised based on their roles as small- and mid-sized organisations (SMOs) or as tenants. The reason participants couldn’t apply for the *Added Value Grant* or the *Farming Investment Fund* was because, again, of the labour-intensive process of applying and the up-front capital required to access the grants. The fact that the *R&D Capital Allowance* and *130% Super-Deduction* are only available to organisations that pay corporation tax is indicative of an assumption that only corporations conduct R&D. Similarly, participants who were running small mills voiced the feeling that regulations like the fortification requirement in the Bread and Flour Regulation (1998) are crafted by policymakers without considering the differential impact on small versus large-scale enterprises. Finally, *apprenticeships* and skills training programmes also fail to develop the types of training pathways that micro-enterprises can participate in.

Exclusion based on the dimension of being a tenant was also raised. The instability of access to land can make it difficult for tenants to invest and scale innovation, particularly for tenant farmers. In designing the new agri-environmental scheme to replace EU farm subsidies, Defra is shortening the commitment term for accessing the *Countryside Stewardship* subsidies to two years rather than five to make it accessible for tenant farmers (Defra, 2023b). However, while this shows a recognition of tenant farmers as an excluded group, it does little to mitigate the larger impact of short farm tenancies on on-farm innovation investments.

Finally, in the case of the *National Planning Policy Framework*, marginalisation was rooted in participants’ roles as specifically O-Ae arable farmers. A livestock farmer would technically be able to get approval for building housing for an additional presence on the farm but industrial arable farms, which run on very little labour and the use of large machinery, would not necessarily need additional help. However, O-Ae farms are more labour-intensive operations and national planning policy does not account for these additional labour needs.

Marginalisation based on being an SMO, O-Ae arable farmer or tenant draws attention to the government’s normative values that benefit large companies and land by default. These values—the sanctity of private property and economic growth—are deeply entrenched and their resultant impact on sustainability transitions is substantial (Clapp 2021). They will need to change if sustainability transitions are to advance. But for the purposes of identifying where grassroots innovators could stretch and transform the regime without being given a protected space, clarifying that marginalisation is targeted at SMOs and tenants helps to identify opportunities for grassroots innovators to nurture links with other sectors with which they could align advocacy efforts, such as tenant farmer unions or SME representative bodies. From an agricultural innovation ecosystems perspective as well as sustainability transition perspectives, identifying these linkages between innovation systems and other social systems reveals where co-benefits can be found and policy incoherence resolved. From a social movements standpoint, these linkages present opportunities for coalition building, where resources, knowledge and support could be pooled together with other sectors to generate small wins that advance the aims of multiple groups at once.

### What (types of innovation)

Regarding the ‘what’ dimension of marginalisation, participants have already made a significant advance in this area. The Seed Marketing Regulation (2011) and the Seeds (National List of Varieties) Regulations (2001) have discriminated against unidentifiable seeds since the 1961 UPOV Convention which installed the DUS framework so there would be an internationally recognised way to accurately allocate plant breeders’ rights. The effective result is that only seeds that can be commercialised can be cultivated and diffused—and only those innovators who have the funds, ability and desire to commercialise it can benefit. The corrosive effects of this linkage between seed innovation and commercialisation on farmer seed systems and plant genetic diversity have been extensive and continues to spread into smallholder and indigenous farming communities around the world (Kloppenburg 2010; Timmermann and Robaey, 2016; Wattnem 2016; Girard and Frison 2018).

However, these participants were able to secure a pathway to legally circulating seeds, removing what was a major block to the diffusion of their innovation. Strategically, participants did not push to dismantle the entire plant breeders’ rights regime, an activity that would have no doubt drawn immense political pushback. Rather, they suggested a *supplementation* to the existing regime, securing the right to conduct a parallel activity, and they were able to do this without altering their underlying values and goals—to reinstitute farmer seed systems and a seed commons—even though these goals are in direct opposition to the regime they are supplementing. In taking this approach, they were able to gain a small win in a critical policy arena that is crucial to their stretch-and-transform agenda (Bryson 1988; Termeer and Metze 2019; Bours et al. 2022; Weick 1984).

### Why (values and outcomes)

The ‘why’—the struggle between values held by the participants and those embodied in specific policies—is the most difficult area of marginalisation to address. It is here that the transformation in values that grassroots movements ultimately want is the most challenging to the existing regime and where grassroots innovators are under the most pressure to fit-and-conform rather than stretch-and-transform.

Interestingly, the two laws related to grassroots innovators’ concerns over being co-opted – the organic labelling law and the bread and flour labelling law—were where the participants’ goal of driving change on farms towards organic and agroecological farming systems was marginalised by the omission of restrictions on the marketing of their innovation. The solution proposed for this by some participants was the need for protections against their socio-technical innovation being undermined by competitors. In essence, they were arguing for the need for a protected space – but this was distinct from the ‘protected spaces’ as described in the strategic niche management literature. Within SNM, the point of a protected space is to incentivise the adoption of a technology by protecting it from market pressure via subsidies, tax breaks, funding, etc. with the main goal being to bring more people on board to that technology. In contrast, the participants were discussing the need for a protected space, not to get more people to start using the technology (although they did want that) but to ensure adoption of the technology is accompanied by adoption of the agricultural and environmental values associated with it.

The historical option for grassroots groups to create such a protected space is to do it themselves through certification (e.g. Fair Trade), some of which become regulated (e.g. organic). These programmes ensure transparency for consumers but are not necessarily the best method for protecting *plurality* (Stirling 2014a). While it helps create clear dividing lines between different sustainability options, it can also make it easier for alternatives to be co-opted by the regime (Smith 2006; Jaffee and Howard 2010). Also, while the diverse grain network could technically attempt to create their own branded ‘stamp of approval’ on certain products, such a solution is not generalisable or scalable. If every grassroots sociotechnical innovation were to have its own certification, it would drive further fragmentation in the market. Here, the paradox of grassroots growth—that in growing, grassroots movements lose what originally made them so disruptive (Smith 2006; Fressoli et al. 2014)—meets a paradox of plurality, how to create an innovation environment where a plurality of solutions exist without losing a sense of order. This paradox is further exacerbated by the plurality that can exist, not only between different pockets of innovation but within. To start discussing certifications is to start placing hard and fast boundaries around what (and who) is ‘in’ and ‘out’—a difficult process that is rife with politics internal to the grassroots movement, as the process of outlining organic certification standards showed (Jaffee and Howard 2010).

So, while the *lack* of quality and labelling standards is a challenge, it does not necessarily hold the solution. While governance is an incredibly important part of fostering plurality in innovation (Stirling 2014a), it is not quite yet clear what specific policies are needed to protect that plurality once it emerges. But for the purposes of how grassroots innovators can engage with government on the issue now—one consideration might be to focus on stretching government towards adopting plurality itself as a normative value in need of protection and encourage a movement away from dichotomous thinking. Ultimately, it might be an achievement in itself to push governments to protect alternatives from the homogenising grind of neoliberalism (Stirling 2014a, b; Smith and Stirling 2018).

## Conclusion

The analysis above shows that (1) there are many different touchpoints along the grassroots-government interface beyond innovation policy, (2) grassroots innovators are marginalised across a range of these policies and (3) there are specific opportunities where grassroots innovators can start to push a *stretch-and-transform* agenda and achieve small wins prior to being granted a protected space by government. These findings contribute to the literature on grassroots innovations and sustainability transitions by highlighting that, while protected spaces are highly beneficial and helpful for cultivating and diffusing grassroots innovations, pursuing them is not the only option available to grassroots niches if they want to have an impact on the regime.

In conducting this analysis, I have proposed a new methodology for mapping policies along the grassroots-government interface and introduced a new method of conceptual analysis for interpreting these policies, drawing on Schillo and Robinson (2017). In introducing this method and analysis, certain aspects were outside the scope of this study that are no doubt critical to gaining a deeper understanding of the mechanics of marginalisation of grassroots movements. Most notably, governance processes and how and when grassroots innovators are marginalised during the policymaking process is crucial to understand (Patterson et al. 2017; Smith and Stirling 2018; Clark et al. 2021; Galli et al. 2020; de Boon et al. 2022) and would be the top priority in pursuing this line of research further, expanding the framework introduced here to include *how* as well as who, what and why. Also, this study focused on national policies, but local and regional policies are incredibly important in the advancement of grassroots movements and further research should delve into these policies further. Building on this, it was clear from discussions with participants that certain policies loom larger than others and that not all policies are equally as impactful on grassroots movements. However, for the purposes of the study, where the goal was to gain a comprehensive view, all policies here are presented in parallel. A next step in the utilisation of Table 2 might be to then develop an advocacy strategy within grassroots movements to identify which policies present both the biggest opportunity for advancing their agenda whilst simultaneously being feasible to impact with the resources available. Finally, while the method of policy mapping employed here revealed a broad range of policies, policies can also change fast and frequently, so what is captured here is necessarily a snapshot rather than a fully comprehensive and contiguous view. Methodological developments of the policy mapping proposed here might focus on how to guarantee both a complete and in-depth but boundaried view of the entire policy landscape.

The findings from this study must also be considered in light of the limitations and challenges that grassroots movements face in utilising their agency and power towards sociotechnical change. Chief among these challenges may be the immense diversity held within grassroots movements about what and how much change is desirable and how this change should be achieved (White and Stirling 2013). For example, some participants, primarily farmers, thought it highly desirable that large supermarket chains start purchasing diverse grains because of the massive market potential and, consequently, the incentive this would create for more farmers to transition to O-Ae farming systems. Others, however, considered the direct trust-based relationships between O-Ae farmers and small artisanal bakers to be a top priority and for involvement with diverse grains to necessarily include this component. These types of disagreements indicate that one of the greatest challenges to operating as a social movement pursuing a strategic policy agenda might not be the political conflicts with vested interests but the internal conflicts within movements. Pinpointing where the challenges are rooted in policy marginalisation versus internal challenges is crucial to identifying where the hurdles lie to stretching and transforming the regime.

Ultimately, however, grassroots innovators are working on cultivating and diffusing their sociotechnical innovations in broad and complex policy ecosystems. Rather than focus on one specific point in this ecosystem—innovation policy—and placing all their efforts on gaining legitimacy within those institutions and waiting to ‘be empowered’ through securing a protected space, there is a wide range of areas in which grassroots innovators may apply their agency and power, met with strategic knowledge, to achieve small wins in pursuit of stretching and transforming food systems.

## Footnotes

1 ‘Heritage grains’ is somewhat of a misnomer as it implies the value of these varieties is in how old they are. Rather, interest in these grains is due to their popularity prior to the widespread use of synthetic inputs, with traits that make them well suited to O-Ae farm systems.

2 It is important to note that genetically diverse grains for O-Ae systems are a ‘new innovation’ compared to the past 70 years of commercial breeding efforts. Informal diversity-led breeding is, indeed, still how seeds are selected and saved by farmers in much of the world today. However, it is discussed here as an innovation in that it has the potential to radically reconfigure existing sociotechnical systems if widely adopted (again) in industrialised countries.
